# Immunotherapy for Glioblastoma: Current Progress and Challenges

**DOI:** 10.3389/fimmu.2021.676301

**Published:** 2021-05-13

**Authors:** Miranda W. Yu, Daniela F. Quail

**Affiliations:** ^1^ Rosalind and Morris Goodman Cancer Research Centre, McGill University, Montreal, QC, Canada; ^2^ Department of Physiology, McGill University, Montreal, QC, Canada; ^3^ Department of Medicine, Division of Experimental Medicine, McGill University, Montreal, QC, Canada

**Keywords:** glioblastoma, brain cancer, immunotherapy, tumor microenvironment, resistance to therapy

## Abstract

Glioblastoma is a highly lethal brain cancer with a median survival rate of less than 15 months when treated with the current standard of care, which consists of surgery, radiotherapy and chemotherapy. With the recent success of immunotherapy in other aggressive cancers such as advanced melanoma and advanced non-small cell lung cancer, glioblastoma has been brought to the forefront of immunotherapy research. Resistance to therapy has been a major challenge across a multitude of experimental candidates and no immunotherapies have been approved for glioblastoma to-date. Intra- and inter-tumoral heterogeneity, an inherently immunosuppressive environment and tumor plasticity remain barriers to be overcome. Moreover, the unique tissue-specific interactions between the central nervous system and the peripheral immune system present an additional challenge for immune-based therapies. Nevertheless, there is sufficient evidence that these challenges may be overcome, and immunotherapy continues to be actively pursued in glioblastoma. Herein, we review the primary ongoing immunotherapy candidates for glioblastoma with a focus on immune checkpoint inhibitors, myeloid-targeted therapies, vaccines and chimeric antigen receptor (CAR) immunotherapies. We further provide insight on mechanisms of resistance and how our understanding of these mechanisms may pave the way for more effective immunotherapeutics against glioblastoma.

## Introduction

Glioblastomas are grade IV gliomas of the central nervous system (CNS) and are the most common and most aggressive type of brain maligancy (1). Patient prognosis is extremely poor, with a median survival of less than 15 months with the current standard of care (SOC), which consists of surgical debulking followed by radiation and chemotherapy (temozolomide) (2). Glioblastomas are currently considered incurable, and all patients inevitably experience and succumb to tumor recurrence, highlighting the urgent need to identify new therapeutic options (3).

The 2016 World Health Organization (WHO) classification of CNS tumors broadly groups glioblastomas based on the mutational status of isocitrate dehydrogenase 1/2 (IDH) (4). Most glioblastomas are IDH-wildtype (wt), which typically arise in older patients (age >50) and are associated with poor prognosis (4). A small subset of glioblastomas (~10%) are IDH-mutant (mut), which are often secondary tumors that arise from the progression of lower grade gliomas and are associated with better survival compared to IDH-wt (4). Glioblastomas can be further classified into classical, mesenchymal, and proneural subtypes based on unique molecular signatures (5, 6). Classical tumors are characterized by EGFR amplification as well as lack of TP53 mutations and homozygous deletion of CDKN2A (5, 6). Mesenchymal tumors have the worst prognosis and are characterized by expression of NF1, often co-mutated with PTEN (5, 6). Proneural tumors have the best prognosis and are characterized by PDGFRA expression (5, 6). Whilst it was previously thought that a fourth subtype (neural) existed, this notion was revised after the neural signature could not be found in tumor cells (5, 6). Glioblastoma tumors are highly heterogenous, with multiple subtypes making up different regions of a single tumor (7, 8). Moreover, each subtype is functionally distinct with unique immunological landscapes including differences in T cell infiltration and macrophage/microglia composition (9). For example, loss of NF1 (i.e. mesenchymal subtype) is associated with a characteristic increase in tumor-associated macrophages (TAMs) (9). Recurrent glioblastomas tend to accumulate macrophages and resemble a mesenchymal state as they become increasingly aggressive and treatment-resistant (10). The immense heterogeneity and microenvironmental evolution of glioblastoma tumors must be considered when developing potential therapies.

Since the addition of temozolomide to glioblastoma SOC in 2005 (2), substantial research efforts and hundreds of clinical trials have been initiated to in an effort to further improve SOC, with very little success. Anti-angiogenic drugs such as bevacizumab, an inhibitor of vascular endothelial growth factor-A (VEGF-A), and cilengitide, an inhibitor of ⍺Vβ3 and ⍺Vβ5 integrin, have been highly pursued in glioblastoma clinical trials, however both of these compounds failed to improve survival of newly diagnosed and recurrent glioblastoma (11–13). In fact, out of the hundreds of clinical trials that have been initiated for glioblastoma in the last decade, few have improved overall survival. Among those that have been moderately successful is the tumor-treating fields (TTF) device, which was approved by the U.S. Food and Drug Administration (FDA) in 2011 for recurrent or refractory glioblastoma (14). TTF involves the local delivery of low-intensity electric fields to disrupt mitosis of glioblastoma cells. In phase III clinical trials, patients with newly diagnosed glioblastoma treated with TTFs in combination with maintenance chemotherapy had a median overall survival of 20.9 months compared to 16 months with maintenance chemotherapy alone (14). Despite this modest success, TTFs have not been incorporated into SOC due to ongoing skepticism amongst the medical community regarding the unblinded nature of TTF trials, as well as issues with patient compliance, which is critical for treatment efficacy (15).

Overall, the failure of past therapeutic candidates to improve glioblastoma SOC is in part a reflection of the rapid and aggressive progression of this disease. Therefore, major research efforts are being made to better understand the brain tumor microenvironment (TME), which holds untapped potential for novel cancer therapies. The immune compartment of glioblastomas is quite substantial, with the majority of cells coming from the myeloid lineage (16). Despite this, glioblastomas are effective at escaping host immune surveillance. Indeed, one of the hallmarks of cancer is the ability to evade cellular immunity (17). Immunotherapies seek to re-direct immune cells against a tumor by exploiting a patient’s immune system. Many immunotherapies such as immune checkpoint inhibitors (ICIs) and chimeric antigen receptor (CAR) T cell therapy have been enormously successful for other aggressive cancers and are now being investigated as potential therapies for glioblastoma (18–22). Herein, we review several ongoing immunotherapeutic approaches for glioblastoma with a focus on ICIs, myeloid-targeted therapies, tumor vaccines, and CAR immunotherapies. We further discuss some key challenges facing immunotherapy in glioblastoma including mechanisms of resistance, which must be overcome in order for the next generation of immunotherapeutics to bring meaningful benefit to patients.

## Immune Privilege and the Central Nervous System: A Case for Immunotherapy

The unique relationship between the brain and the immune system is central to the use of immunotherapy in brain diseases such as glioblastoma. Historically, the brain has been viewed as a tightly sealed organ, guarded by a closely regulated blood brain barrier (BBB), and devoid of any lymphatics or immune surveillance. However, this notion of “immune privilege” was disputed when it was discovered that allo-antigens could illicit an immunological response in the brain (23). Several subsequent isograft versus allograft studies further substantiated this field-shifting discovery (24, 25). As a result of technological advances such as intravital imaging, it is now known that immune surveillance and specifically, the priming and activation of T cells, largely takes place in the meningeal compartment of the CNS (26). However, it only became clear in the last decade how the CNS connects to the peripheral immune system. In 2015, two seminal studies showed for the first time a network of functional lymphatic vessels that line the dural sinuses, which drain into the deep cervical lymph nodes, and serve as a gateway for T cell trafficking between the periphery and the cerebrospinal fluid (CSF) of the CNS (27, 28). While once thought to be immune privileged, it is now appreciated that the brain receives constant immune surveillance and communication with the peripheral immune system, allowing the possibility of immunotherapy as a means of treating diseases of the CNS.

Despite these potential opportunities, one remaining challenge for glioblastoma treatment efficacy is overcoming the BBB. This tightly regulated barrier between the peripheral blood and CNS functions to facilitate the movement of ions, neurotransmitters, and nutrients while shielding the CNS from neurotoxins and most macromolecules (29). Thus, while small (<400Da), lipid-soluble (<8 hydrogen bonds) drugs may be able to passively diffuse across the BBB (30), large or water-soluble drugs are largely excluded by a network of extremely tight junctions (29). This presents a significant challenge for systemic immune-based therapies that rely on effective antibody delivery into tumors or peripheral transfer of cells. Interestingly, one of the hallmarks of brain tumors is a loss of BBB integrity and subsequent increased tight junction permeability (31). In glioblastoma, this characteristic is attributed to loss of claudin-3 and altered levels of claudin-1 and claudin-5, which are the major structural proteins that regulate BBB tight junction permeability (32, 33). While disruption of the BBB may seemingly be advantageous for drug delivery, especially for drugs that depend on the recruitment of peripheral immune cells, loss of BBB integrity may also enhance tumorigenicity by enabling the infiltration of pro-tumorigenic cells such as peripherally-derived immunosuppressive macrophages (34). This double-edged sword is further complicated by the fact that the BBB is not ubiquitously disrupted, and in fact remains completely intact within specific regions of glioblastoma tumors (35). Therefore, systemic therapies for glioblastoma must be able to overcome these complex limitations in order to be effective.

### Immune Checkpoint Inhibitors (ICIs)

**Figure 1:**
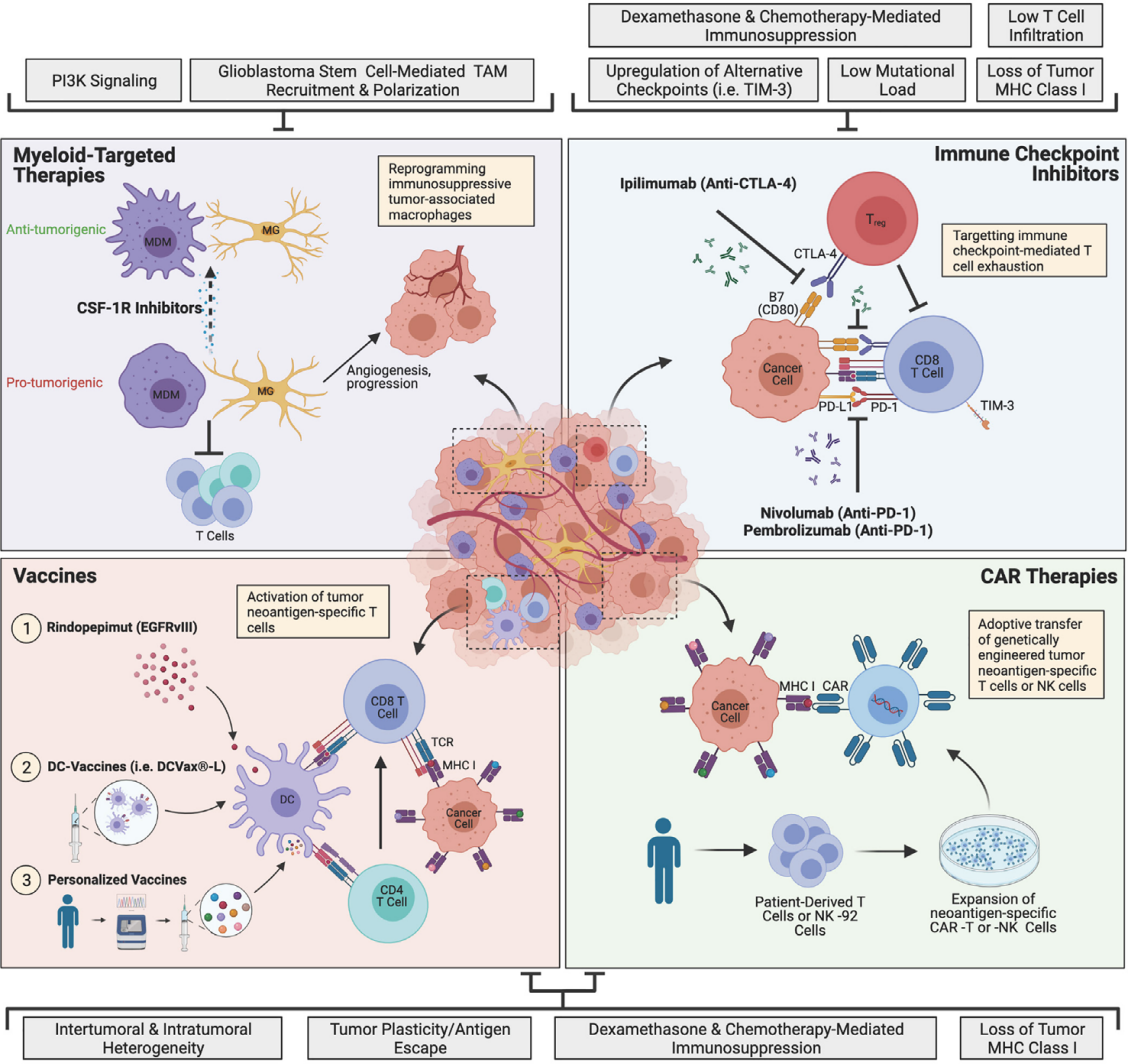
The current landscape of major glioblastoma immunotherapies and mechanisms of resistance. Immune checkpoint inhibitors (ICIs) target T cell exhaustion through blockade of immune checkpoints PD-1 and CTLA-4 to restore T cell function and antitumor activity. Myeloid-targeted therapies such as CSF-1R inhibitors reprogram immunosuppressive microglia (MG) or monocyte-derived macrophages (MDMs) (pro-tumorigenic) to become more anti-tumorigenic. Peptide vaccines, dendritic cell (DC)-vaccines and personalized vaccines educate T cells to target tumor neoantigen(s). Chimeric antigen receptor (CAR) immunotherapies involve genetically engineering a patient’s own T cells or non-patient NK-92 cells to express neoantigen-specific CARs, which are expanded in culture and adoptively transferred to the patient. Glioblastoma is highly resistant to therapy, and currently, none of the depicted immunotherapies have succeeded in improving treatment, although many clinical trials are currently ongoing. The grey boxes outline major mechanisms of resistance that are barriers to each immunotherapeutic approach, including intrinsic, adaptive and iatrogenic mechanisms. Image made with BioRender.com.

The discovery of immune checkpoint molecules PD-1 and CTLA-4 has undoubtedly transformed the field of cancer immunotherapy (36, 37). Anti-CTLA-4 and anti-PD-1/PD-L1 ICIs have been extremely successful for aggressive cancers such as advanced melanoma and non-small cell lung cancer (NSCLC) (18–20), and there is growing interest in the utility of ICIs as a potential treatment for glioblastoma. In chronic inflammatory conditions such as cancer, prolonged T cell activation leads to increased CTLA-4-expressing T_regs_ and upregulation of CTLA-4 on cytotoxic T lymphocytes (CTLs), which interacts with the B7 family of receptors and leads to reduced T cell proliferation and survival (**Figure 1**) (38). In gliomas, this immunosuppression is bolstered by the upregulation of PD-L1 on tumor cells and circulating monocytes/macrophages, which further inhibits CD8^+^ and CD4^+^ T cell activation (39, 40). Prolonged T cell activation also causes upregulation of PD-1, which recognizes PD-L1 on antigen-presenting cells (APCs) and tumor cells, and results in T cell exhaustion and reduced survival (**Figure 1**) (38). These immune signatures, including the upregulation of multiple immune checkpoints and an increased fraction of T_regs_, are highly characteristic of the glioblastoma TME, and warrant investigation of ICIs as a potential means of restoring T cell responses (41–44).

Accordingly, several studies have explored the use of ICIs in experimental models of glioma and results have been promising (45–49). For example, in an implanted mouse model of glioma using SMA-560 cells, anti-CTLA-4 conferred long-term survival in 80% of mice, and reduced the fraction of infiltrating T_regs_ (49). Additionally, anti-PD-1 eradicated 44% of orthotopic GL261 tumors when used alone, and 100% when combined with temozolomide (45). In a glioblastoma stem cell (GSC) mouse model, triple combination therapy with anti-CTLA-4, anti-PD-1 and an IL-12 expressing oncolytic virus (G47Δ-mIL12) cured 89% of mice, with 100% of the cured mice alive at 96 days post-tumor re-challenge, suggesting establishment of immunological memory with this combination therapy (50).

Although preclinical work has been promising, ICI efficacy in glioblastoma patients has been limited. There have been a number of case studies reporting dramatic responses in glioblastoma patients receiving nivolumab (anti-PD-1) (51, 52), most striking of which is the case of a 60-year-old patient with recurrent glioblastoma who received nivolumab for 2 years without any progression, toxicity or need for corticosteroid treatment (52). Despite these exceptional cases, overall, ICI clinical trials in glioblastoma have been disappointing. Checkmate 143 trial was the first randomized trial testing ICIs for recurrent cases of glioblastoma. The initial phase I study assessed the safety of nivolumab (anti-PD-1) and ipilumamab (anti-CTLA-4) in 40 patients with recurrent disease, and results showed that nivolumab alone was better tolerated compared to the dual therapy, with adverse advents associated with ipilumumab (53). Unfortunately, the subsequent open-label randomized phase 3 trial comparing nivolumab to bevacizumab failed to improve overall survival in 369 patients with recurrent glioblastoma (54). Additionally, a recent phase II clinical trial assessing pembrolizumab (anti-PD-1) with or without bevacizumab in recurrent glioblastoma patients failed to meet the primary endpoint of 6 months progression-free survival (PFS) with either therapeutic approach (55). Attention has since shifted to newly diagnosed glioblastoma, where a pre-surgical dose of nivolumab followed by post-surgical continuation of treatment was reported to provide long-term survival benefit in two patients with newly diagnosed glioblastoma, who were alive at 33 and 28 months post-surgery (56). However, all clinical studies to-date evaluating nivolumab in primary glioblastoma, including Checkmate 498 and Checkmate 548 trials, have failed to meet primary endpoints.

Overall, ICIs have failed to demonstrate a significant benefit in glioblastoma thus far and several explanations have been proposed (**Figure 1**). Glioblastomas are inherently immunologically “cold”, containing few T cells and predominantly occupied by pro-tumorigenic TAMs, particularly in IDH-wt tumors (57, 58). While ICIs may initially restore T cell function, the overwhelming presence of immunosuppressive myeloid cells remains a prevailing source of resistance to treatment (59). Immunologically “hot” tumors, characterized by high T cell infiltration and immune activation, have generally been more responsive to ICIs, and there is ongoing research aimed at understanding how to turn immunologically cold tumors, like glioblastoma, into hot tumors, in order to improve ICI efficacy (60, 61). Moreover, only 3.5% of glioblastomas exhibit a high tumor mutational load (62), which influences sensitivity to ICIs (63), suggesting that a very small minority of glioblastoma patients are likely to benefit from this treatment.

Another potentially overlooked mechanism of resistance to ICIs is iatrogenic resistance in response to chemotherapy or steroids. The combination of ICIs with chemotherapy is receiving widespread attention as a mechanism to induce tumor mutations (neo-antigens) (64). However, systemic chemotherapy, including temozolomide, is inherently immunosuppressive and causes lymphodepletion and myelotoxicity in preclinical models and in cancer patients (64). This may be particularly harmful for glioblastoma patients as tumor-infiltrating lymphocytes are already rare. Studies have explored the possibility of local chemotherapy using implanted slow-release polymers (65, 66), which avoids systemic lymphodepletion and significantly enhances response to ICIs in preclinical models by increasing tumor antigen-specific T cells (67). In addition, corticosteroids are routinely prescribed for cancer patients to manage symptoms, including dexamethasone, which is given to glioblastoma patients to manage cerebral edema. However, corticosteroids are anti-inflammatory, and may antagonize the therapeutic effects of ICIs; in fact, they are used to treat immune-related adverse events from ICIs (68). Alternative therapies for cerebral edema have been proposed, such as bevacizumab or mannitol. However, both agents come with significant drawbacks, including the need for repeated intravenous infusions, elevated bleeding risk (69), impaired perioperative healing (69), hypertension (70), and diminished efficacy with prolonged use (71). Therefore, it is unclear how to effectively integrate ICIs with current SOC treatments that are critical for glioblastoma management.

Finally, glioblastoma tumors can adapt to immune checkpoint blockade by upregulating alternative checkpoints such as TIM-3 following ICI treatment (72). Combining anti-PD-1 with TIM-3 blockade may potentially overcome this acquired resistance. For example, combining anti-PD-1 with anti-TIM-3 improved overall survival from 28% (anti-PD-1 alone) to 60% (dual therapy) in preclinical GL261 models, and this was further enhanced to 100% when combined as a triple therapy with stereotactic radiosurgery (SRS) (73). In addition to the PD-1 pathway, recent work has identified expression of the inhibitory receptor CD161 on intratumoral T cells in glioblastoma, and blockade of CD161 enhanced T cell anti-tumor activity both *in vitro* and in GL261 transplantable mouse models (74). Interestingly, CD161 is encoded by the NK cell gene, *KLRB1*, highlighting NK cell receptors as potential targets for immunotherapy. Taken together, future studies should explore novel targets and combination therapies to improve ICI efficacy.

### Myeloid-Targeted Therapies

Macrophages are the most abundant cell type in glioblastoma, accounting for up to 30% of the tumor, and are highly associated with disease progression (16, 75). In glioblastoma, macrophages can be either yolk sac-derived tissue-resident microglia (MG) or monocyte-derived macrophages (MDMs) from the periphery (34, 76, 77), with infiltrating MDMs representing the majority of TAMs (78). In addition to having distinct ontogenies, TAMs also adopt a variety of activation states that are not restricted to the conventional M1/M2 designations (77, 79). Interestingly, glioblastoma stem cells (GSCs) have been shown to recruit TAMs by secreting periostin and cytokines associated with alternative activation (80, 81). Once recruited, TAMs further drive disease progression by enhancing the invasion of GSCs through TGF-β1 signaling (82). In addition to the direct protumorigenic effects of TAMs, they can also indirectly mediate tumor progression by promoting T cell exhaustion *via* the PD-L1/PD-1 pathway (**Figure 1**) (83). Moreover, infiltrating TAMs in glioblastoma lack essential costimulatory molecules for T cell activation (CD80, CD86, CD40), which further contributes to an immunologically inactive tumor (84). Finally, TAMs play an important role in tumor angiogenesis and have been associated with resistance to anti-angiogenic therapies such as bevacizumab (**Figure 1**) (85–87). Angiogenic factors not only facilitate tumor progression, but also suppress APCs, DCs and T cells, while augmenting the effects of TAMs and T_regs_, resulting in a continuous cycle of immunosuppression (88). Taken together, therapies that target the myeloid compartment may be an effective approach to reversing active immunosuppression in the TME and preventing tumor progression.

There are many approaches to targeting TAMs in glioblastoma, one of which is inhibition of colony stimulating factor 1 receptor (CSF-1R), an important receptor for macrophage differentiation and survival (89, 90). In mice, CSF-1R inhibition re-educates macrophages to adopt an anti-tumor phenotype, leading to tumor regression and increased survival, with a particularly profound effect in proneural glioblastoma (89, 90). However, despite dramatic improvements in survival, drug resistance eventually develops *via* alternative pathways such as PI3K signaling (**Figure 1**) (91). In a phase II clinical study, treatment with CSF-1R inhibitors in recurrent glioblastoma patients failed to meet primary endpoint of 6 months PFS (92), which may be attributable to the high frequency of PTEN and PI3K pathway mutations among glioblastoma patients (5, 93). Although CSF-1R inhibitors have generated little clinical success as monotherapies, emerging studies have suggested that TAM-targeted therapies may be synergistic with radiotherapy, which may serve as a more effective approach for targeting the myeloid compartment (94, 95). In GL261-implanted glioblastoma mice, irradiation enhanced survival when combined with local delivery of lipid nanoparticles directed against PD-L1-expressing TAMs and dinaciclib, a cyclin-dependent kinase 5 inhibitor (95). Moreover, in preclinical mouse models of glioblastoma driven by PDGFB overexpression and/or p53 knockdown, irradiation combined with daily CSF-1R inhibition drastically increased survival compared to either treatment alone (94). Despite these promising preclinical studies, a phase 1b/2 clinical trial evaluating CSF-1R inhibition in combination with radiotherapy and temozolomide for newly diagnosed glioblastoma did not improve median PFS or overall survival compared to historical controls (NCT01790503) (96). Although a comprehensive review of why this clinical trial failed is currently ongoing, preclinical studies demonstrated that daily dosing was critical to the efficacy of CSF-1R inhibition and unfortunately, patient tolerability restricted dosing to 5 days/week in the clinical setting (94).

In contrast to CSF-1R inhibitors, which target bulk macrophages, little is known about the potential benefit of targeting specific macrophage phenotypes and/or their recruitment. New studies have enabled the investigation of MG and MDMs and their distinct contributions to glioblastoma based on identifying distinguishing markers such as MDM-specific expression of CD49d and expression of Tmem119, CX3CR1 and SiglecH on MG (34, 97, 98). In accordance with these findings, anti-CD49d has been shown to selectively reduce tumor MDM numbers in preclinical glioblastoma models (94). Interestingly, while anti-CD49d monotherapy had no impact on survival, combining this treatment with irradiation prolonged survival in both mouse models, warranting further investigation (94). In the GL261 mouse model of glioblastoma, histological analyses have shown that MDMs are more readily recruited to perivascular tumor regions compared to MG, which is a niche for GSCs (78). Moreover, selectively limiting MDM infiltration through genetic *Ccl2* reduction prolongs survival of GL261 tumor-bearing mice (78). Although targeting CCL2-mediated recruitment of MDMs has not yet been clinically explored, combining CCL2 inhibition with anti-PD-1 treatment prolonged survival in GSC glioblastoma-bearing mice, and may be a potential candidate for future studies (99). Interestingly, Tie2-expressing MDMs have been identified as a distinct hematopoietic lineage of cells that are actively recruited to glioblastoma tumors and were shown to drive tumor angiogenesis in an orthotopic xenograft model of human glioblastoma (87). Remarkably, loss of Tie2-expressing MDMs completely abrogated neovascularization in human glioblastoma-derived tumor-bearing mice, suggesting that selectively targeting Tie2-expressing MDMs may be another potential therapeutic avenue (87). Taken together, reprogramming macrophage phenotypes and targeting specific TAM recruitment may be a more effective approach to disease control that has yet to be clinically explored.

### Vaccines

Oncogenic driver mutations and passenger mutations can give rise to new proteins (neoantigens), which contain unique sequences (neoepitopes) that can be recognized by T cells when presented by major histocompatibility complex (MHC) molecules on the surface of cancer cells or APCs (100). Vaccine-based therapeutics facilitate the education of tumor-specific CTLs by soliciting highly expressed tumor neoepitopes (**Figure 1**) (101). The most rudimentary approach to therapeutic vaccines is to directly administer one or more peptides that mimic the tumor neoepitope(s) of interest, although dendritic cell (DC)-based vaccines and personalized vaccines are also being explored as potential therapies in glioblastoma.

Approximately 40% of glioblastomas overexpress *EGFR*, with the most common variant being EGFRvIII, arising from the loss of exons 2-7 from the *EGFR* coding sequence (102–104). The high frequency of EGFRvIII across glioblastoma patients has led to the development of Rindopepimut (CDX-110), a synthetic 14-amino acid peptide that mimics the EGFRvIII mutational site coupled to keyhole limpet hemocyanin (KLH), an immunogenic carrier protein (105). In 2015, the FDA granted rindopepimut the “Breakthrough Therapy Designation”, supporting the expedition of its approval for glioblastoma, given that clinical studies demonstrate substantial benefit over other available therapies. The single arm multicenter phase II trial (ACT III), which administered rindopepimut and adjuvant chemotherapy for newly diagnosed EGFRvIII^+^ glioblastoma patients, had promising results with a median overall survival of 21.8 months compared to matched historical controls treated with SOC (106). However, the subsequent randomized double-blinded phase III trial (ACT IV) failed to demonstrate any increase in survival and was terminated (107). Loss of EGFRvIII expression following vaccination suggests that the recurrent tumor can become resistant to EGFRvIII-targeting memory T cells (**Figure 1**) (106). In fact, half of all glioblastomas that are initially EGFRvIII^+^ lose EGFRvIII expression upon recurrence (108). While overexpression of EGFRvIII was once believed to be predictive of poor prognosis (103), a recent study assessing the EGFR status of 106 patients found no association between EGFRvIII and overall survival or progression-free survival in either newly diagnosed or recurrent glioblastoma (104). Taken together, these observations may explain why EGFRvIII-targeted vaccines have failed to control disease and improve survival.

DCs are an essential component of vaccination because of their role in antigen presentation and the priming and activation of T cells (101). It was once thought that DCs played little to no role in the active immunity of the brain, with MG assumed to be the predominating APCs (109, 110). However, DCs are increasingly being recognized for their functional role in the brain as APCs and it has been reported that they can even arise from MG differentiation (111, 112). Interestingly, MG exhibit a great amount of plasticity and can be skewed towards macrophage-like or DC-like cells by M-CSF or GM-CSF, respectively (111). While traditional vaccines rely on the activation of DCs and other APCs *in vivo*, DC-based vaccines deliver DCs pre-loaded with antigen by pulsing patient-derived DCs *ex vivo* with either tumor lysate or predetermined neoantigens (101). For glioblastoma, DC-based vaccines have shown promise in early clinical studies (113, 114). A phase 1 clinical study investigating the dose-escalation of DCs pulsed with tumor peptides in 12 newly diagnosed glioblastoma patients demonstrated safety and tolerability of this therapy (113). The double-blinded randomized phase II trial of ICT-107, involving DCs pulsed with six synthetic peptides, increased overall survival of newly diagnosed glioblastoma patients by 2 months compared to placebo control, although it was not statistically significant (114). Another DC vaccine, DCVax^®^-L, demonstrated safety and tolerability in early studies and recently underwent phase 3 evaluation, but was unfortunately prematurely suspended due to lack of funds (115). Interestingly, there appears to be subtype-specific benefits of DC-based vaccines, whereby the mesenchymal subtype is associated with heightened responsiveness, including increased infiltration of CD3^+^ and CD8^+^ T cells compared to other glioblastoma subtypes, and increased survival compared to historical controls of the same molecular subtype (116). Therefore, molecular subtyping may be an important consideration for future study enrollment and design.

Neoantigen-targeted vaccines for glioblastoma are extremely limited by the high level of inter- and intra-tumoral heterogeneity of these tumors (**Figure 1**) (7, 8). Tumor cells also actively evade T cell immunosurveillance by altering surface MHC expression and antigen presentation pathways (**Figure 1**) (117). Thus, while the identification of neoantigens is critical, immunization against a single molecular target, such as EGFRvIII (rindopepimut), selectively eliminates neoantigen-expressing cells, leaving the remaining tumor resistant to the activated T cells (106, 118). As an alternative approach, personalized vaccines may be more appropriate in highly heterogenous tumors like glioblastoma (100). The personalized vaccine pipeline involves first characterizing the mutational profile of an individual’s tumor through comparative sequencing, followed by selection of patient-specific targets and finally, vaccine production (100). This personalized approach effectively circumvents patient-to-patient variability and seeks to maximize the affected tumor area by generating T cell immunity against many targets. Preliminary studies using personalized vaccines in newly diagnosed glioblastoma patients have been generally positive (119, 120). In a phase I/Ib trial, patients were immunized post-radiation with up to 20 synthetic long peptides generated based on tumor DNA/RNA sequencing, and given an immunostimulant, poly-ICLC. Neoantigen-specific T cell responses were observed in patients who did not receive dexamethasone and multiplex immunofluorescent staining of tumor specimens revealed increased CD8^+^ and CD4^+^ T cell infiltration in these responsive patients (119). Combining personalized neoantigen vaccines with vaccination against unmutated antigen (GAPVAC) have shown similarly promising results where immunization generated sustained central memory CD8^+^ T cell responses against unmutated antigen, as well as neoepitope-specific Th1 responses in CD4^+^ T cells (120). There are currently over 50 ongoing clinical trials for various forms of vaccines against glioblastoma, with results expected to be rolled out in the coming years.

### CAR Immunotherapies

CAR T cell therapy is a highly personalized form of adoptive T cell therapy that takes advantage of a patient’s own T cells and strategically engineers them to express CARs, which target cancer cells (**Figure 1**). CARs consist of an intracellular T cell activation domain and an extracellular antigen-recognition domain, which are joined together by a transmembrane domain connected to a hinge (121). For refractory hematologic cancers such as acute lymphoblastic leukemia (ALL) and diffuse large-B-cell lymphoma (DLBCL), CAR T cell therapy has been transformational (21, 22), however translating this therapy to solid tumors comes with a unique set of challenges and no CAR T cells have been approved for solid cancers to-date (122). Since their inception, CARs have quickly evolved from basic CD3ζ-signaling in the first-generation, to incorporating co-stimulatory domains such as CD28, 4-1BB, OX40 and ICOS in second and third-generations, followed by the addition of cytokine-expressing domains in fourth-generation CARs (TRUCKs) and most recently, cytokine receptor-expressing domains in fifth-generation CARs (121–123). Despite the successful engineering of more potent and immunogenic CAR-T cells, off-target effects, poor tumor infiltration and a highly immunosuppressive TME remain major barriers to the clinical efficacy of CAR T cells for solid tumors (121).

There are several ongoing CAR T cell candidates for glioblastoma including CARs directed against EGFRvIII, IL13Rα2 and HER2. In an orthotopically transplanted human glioblastoma xenograft model, third generation EGFRvIII-specific CAR T cells prolonged survival of tumor-bearing mice by up to 55 days compared to untreated mice (124). However, clinical benefit has yet to be observed in patients where tumor adaptations, including loss of EGFRvIII expression and post-treatment infiltration of T_regs_, invariably leads to resistance against EGFRvIII-directed CARs (118, 125). Alternatively, CAR T cell therapies can target IL13Rα2, which is overexpressed in 58% of glioblastomas and is associated with poor prognosis and a mesenchymal gene signature (126). IL13Rα2-specific CAR T cells have been clinically well-tolerated, and structurally optimized to prevent off-target Fc interactions (127, 128). This therapeutic candidate, which is currently being clinically evaluated (NCT02208362) (129), was reported to cause dramatic tumor reduction and a sustained complete clinical response (7.5 months) in a patient bearing seven highly aggressive recurrent glioblastoma tumors (128). HER2-targeted CAR T cells have demonstrated similar promise in early phase clinical trials, where careful engineering has improved tumor-specificity and reduced off-target effects (130, 131).

The propensity for glioblastoma tumors to quickly adapt through antigen escape remains a major barrier to CAR T cell therapy (**Figure 1**) (132). To minimize the risk of treatment resistance, it is likely that CAR T cells should target multiple antigens or be combined with a synergistic therapy. For example, a bispecific CAR molecule directed against both IL13Rα2 and HER2 (TanCAR) has been shown to promote tumor regression and increase survival in mice xenografted with a HER2^+^ IL13Rα2^+^ human glioblastoma cell line compared to CAR T cells against either target alone (133). IL13Rα2 CAR T cells are also currently being clinically evaluated in combination with nivolumab and ipilimumab for recurrent and refractory glioblastoma (NCT04003649) (134). Synergistic combinatorial approaches will be instrumental in improving CAR T cell efficacy, since CAR T cells alone have shown limited utility against solid tumors, including glioblastoma, thus far.

As CAR T cell therapy continues to advance, CAR-NK cell therapy has also gained attention as a potential tool for cancer immunotherapy. In glioblastoma, NK cells can mediate tumor cell killing and are associated with good prognosis (135). A notable advantage of CAR-NK cell therapy is the ability to be administered to an HLA-mismatched patient, thus allowing the possibility of an off-the-shelf therapy (136). However, the time and cost associated with NK cell expansion and manufacturing remain a barrier for CAR-NK cell therapy (137). Currently, NK-92 cells are the only NK cell line approved by the FDA and are compliant with good manufacturing practices (138). Remarkably, preclinical testing of HER-2-specific NK-92 cells (NK-92/5.28.z) in an orthotopic xenograft mouse model of glioblastoma demonstrated a dramatic increase in survival (200.5 days) compared to mice treated with control NK-92 cells (73 days) (139). Intracranial injection of NK-92/5.28.z cells are being evaluated in the ongoing CAR2BRAIN clinical trial for recurrent glioblastoma, with no toxicities reported thus far at three dose levels (NCT03383978) (140, 141). Although the field of CAR-NK cell therapy is still relatively new, preliminary results have been promising, and the first ever clinical trial of CAR-NK cells for glioblastoma will indeed shed light on whether this immunotherapy can bring benefit to patients.

## Conclusions

The field of cancer immunotherapy is rapidly evolving to meet the unique requirements and challenges of diverse cancer types. While immunotherapies have revolutionized the clinical management of NSCLC, melanoma, renal cancer, and several hematological malignancies, it is becoming increasingly apparent that mechanisms of efficacy are not one-size-fits-all. For glioblastoma, conventional therapies provide limited benefit to patients and most attempts to incorporate immunotherapeutics have been futile thus far. Efforts to optimize immunotherapies need to overcome many obstacles to achieve efficacy, including physical barriers to drug delivery (e.g. BBB), prominent tumor heterogeneity, abundant GSC niches, lymphocyte scarcity, and the immunosuppressive effects of SOC treatments. Studying the dynamics of different glioblastoma subtypes, as well as long-term survivors, will be an important resource in understanding aspects of the TME that promote survival. Finally, a prevailing challenge in glioblastoma research is that the effects of immunotherapy in animal models rarely recapitulate clinical observations. Genetically-engineered and transplantable mouse models are the best tools available, however, they fail to fully reflect tumor heterogeneity and host antitumor immunity. Further efforts are needed to generate preclinical models that more accurately recapitulate human disease.

Taken together, there is a desperate need to identify new therapeutic opportunities in glioblastoma in order to improve SOC. While immunotherapies have the potential to transform glioblastoma treatment, many are limited by the unique and challenging characteristics of the tumor. With a better understanding of glioblastoma TME dynamics and improved preclinical tools, we can open doors for more personalized and targeted treatments that ultimately have the potential to have a meaningful impact on patient outcomes.

## Author Contributions 

MY and DQ conceived, wrote and edited the manuscript. All authors contributed to the article and approved the submitted version.

## Funding

DQ is funded by the Brain Tumor Funders’ Collaborative (BTFC), Canada Foundation for Innovation (CFI-JELF, 37488), and a Tier II Canada Research Chair in Tumor Microenvironment Research. MY is supported by the Max E. and Jane K. Childress Fellowship from the Department of Physiology, McGill University.

## Conflict of Interest

The authors declare that the research was conducted in the absence of any commercial or financial relationships that could be construed as a potential conflict of interest.
