# Erratum: Immunotherapy for Glioblastoma: Current Progress and Challenges

**DOI:** 10.3389/fimmu.2021.782687

**Published:** 2021-10-07

**Authors:** Frontiers Production Office

**Affiliations:** Frontiers Media SA, Lausanne, Switzerland

**Keywords:** glioblastoma, brain cancer, immunotherapy, tumor microenvironment, resistance to therapy

Due to a production error, there was an error in the Article Title. The final “s” of “Challenges” was missed from the end of the title.

The publisher apologizes for this mistake. The original version of this article has been updated.

